# Rainbow Trout Red Blood Cells Exposed to Viral Hemorrhagic Septicemia Virus Up-Regulate Antigen-Processing Mechanisms and MHC I&II, CD86, and CD83 Antigen-presenting Cell Markers

**DOI:** 10.3390/cells8050386

**Published:** 2019-04-27

**Authors:** Ivan Nombela, Ricardo Requena-Platek, Byron Morales-Lange, Veronica Chico, Sara Puente-Marin, Sergio Ciordia, Maria Carmen Mena, Julio Coll, Luis Perez, Luis Mercado, Maria del Mar Ortega-Villaizan

**Affiliations:** 1 Instituto de Investigación, Desarrollo e Innovación en Biotecnología Sanitaria de Elche (IDiBE) and Instituto de Biología Molecular y Celular (IBMC), Universidad Miguel Hernández (UMH), 03202 Elche, Spain; nombela@umh.es (I.N.); ricardorequena.p@gmail.com (R.R.-P.); vchico@umh.es (V.C.); spuente@umh.es (S.P.-M.); luis.perez@umh.es (L.P.); 2 Instituto de Biología, Pontificia Universidad Católica de Valparaiso, 2373223 Valparaiso, Chile; byron.morales@pucv.cl (B.M.-L.); luis.mercado@pucv.cl (L.M.); 3 Unidad de Proteómica, Centro Nacional de Biotecnología (CNB- CSIC), 28049 Madrid, Spain; sciordia@cnb.csic.es (S.C.); mcmena@cnb.csic.es (M.C.M.); 4 Instituto Nacional de Investigación y Tecnología Agraria y Alimentaria (INIA), 28040 Madrid, Spain; juliocoll@inia.es

**Keywords:** rainbow trout, erythrocytes, red blood cells, VHSV, transcriptome, proteome, antigen presentation, autophagy, ubiquitination

## Abstract

Nucleated teleost red blood cells (RBCs) are known to express molecules from the major histocompatibility complex and peptide-generating processes such as autophagy and proteasomes, but the role of RBCs in antigen presentation of viruses have not been studied yet. In this study, RBCs exposed ex vivo to viral hemorrhagic septicemia virus (VHSV) were evaluated by means of transcriptomic and proteomic approaches. Genes and proteins related to antigen presentation molecules, proteasome degradation, and autophagy were up-regulated. VHSV induced accumulation of ubiquitinated proteins in ex vivo VHSV-exposed RBCs and showed at the same time a decrease of proteasome activity. Furthermore, induction of autophagy was detected by evaluating LC3 protein levels. Sequestosome-1/p62 underwent degradation early after VHSV exposure, and it may be a link between ubiquitination and autophagy activation. Inhibition of autophagosome degradation with niclosamide resulted in intracellular detection of N protein of VHSV (NVHSV) and p62 accumulation. In addition, antigen presentation cell markers, such as major histocompatibility complex (MHC) class I & II, CD83, and CD86, increased at the transcriptional and translational level in rainbow trout RBCs exposed to VHSV. In summary, we show that nucleated rainbow trout RBCs can degrade VHSV while displaying an antigen-presenting cell (APC)-like profile.

## 1. Introduction

Nucleated red blood cells (RBCs) can develop immune responses to viruses that directly target these cells, such as infectious salmonid anemia virus (ISAV) [1] and piscine orthoreovirus (PRV) [2,3,4,5,6], which mainly results in the up-regulation of the interferon (IFN)-α gene and interferon-stimulated genes. Recently, we reported that rainbow trout RBCs can mount an antiviral response against viral hemorrhagic septicemia virus (VHSV) [7]. Also, we have reported that RBCs can be stimulated by infectious pancreatic necrosis virus (IPNV), where up-regulation of IFN type 1-related genes leads to expression of antiviral myxovirus resistance protein Mx [8]. However, rainbow trout RBCs are nonpermissive to VHSV and IPNV infections, and the cellular mechanisms that make the infection nonpermissive are being studied [9]. 

Autophagy is an evolutionarily conserved mechanism in which intracellular material is enveloped in double-membrane vesicles and targeted for fusion with lysosomes for degradation. Numerous pathogens have been known to cause autophagy, including viruses [10]. The role of autophagy in the context of viral infections is still controversial and can have either antiviral or proviral functions depending on the virus and host cell [11]. Autophagy can contribute to the innate immune response by delivering viral pathogen-associated molecular pattern (PAMPs) to endosomal Toll-like receptors (TLRs) [12,13] through vesicle trafficking. Related to VHSV, it was found that rhabdoviral infections, including VHSV, can be inhibited when autophagy is activated [14]. Moreover, the viral glycoprotein G is sufficient to induce autophagy [14] and a Pepscan technique has successfully identified the peptides involved in autophagy activation [15]. In teleosts, VHSV infection in turbot RBCs led to expression of NK-lysin, an antimicrobial peptide, associated with LC3 protein in autophagosomes [16].

Recently, groups have reported on selective autophagy mechanisms, suggesting that autophagy is far from being a nonselective degradative process [17]. Autophagy uses adaptors known as SLRs (sequestosome 1/p62-like receptors) that can selectively target pathogens for degradation in autophagosomes [18]. p62 contains domains that interact with both ubiquitinated proteins and autophagy-specific light chain 3 (LC3) modifier [19] in the inner face of the autophagosome; in this way, p62 is involved in delivering ubiquitinated proteins marked for proteasome degradation to autophagosomes. Ubiquitination is a process mediated by the E3 ligases, in which a series of three different enzymes are involved in the activation, conjugation and ligation of ubiquitin to the proteins targeted for degradation [20]. Ubiquitinated proteins are primarily degraded by the proteasome. The ubiquitin-proteasome system (UPS) plays an important role in cell homeostasis by ensuring the quality of newly synthetized proteins and the regulation of levels of proteins performing critical functions in the cell. Functional 20S proteasomes have been identified in human [21] and rainbow trout [7] RBCs. As with autophagy, the UPS plays a double role in the context of viral infections: it can be manipulated by viruses to bypass host defenses mechanisms or participate in the elimination of viral components [22]. The UPS has been named as the principal source of antigenic peptides for the major histocompatibility complex (MHC) of the immune system [23]. Autophagy is also known to be involved in antigen degradation and delivery to MHC class I and II molecules, which could trigger the adaptive immune response [24,25,26]. 

Antigen presentation is a key process to activate T cells. This process is mediated by antigen-presenting cells (APCs) such as dendritic cells (DCs). DCs act as an important link between the innate and adaptive immune responses and are involved in patrolling tissues, pathogen engulfment, degradation, movement to lymphoid tissues, and T cell stimulation. However, the presence of APCs, and specifically DCs, was largely unknown in fish until recently, when a subset of APCs resembling those of mammals was identified in zebrafish [27] and rainbow trout [28]. APCs are characterized through cell markers such as CD86 and CD83, which serve as costimulatory molecules, and MHC molecules. Among them, MHC molecules are some of the most important proteins involved in the antigen presentation process, as they display pathogen-derived fragments on the cell surface to allow recognition by T cells. Expression of MHC molecules indicates that a cell can play an APC role. MHC class I (MHCI) protein expression has been detected in rainbow trout RBCs [29,30] and MHC class II (MHCII) transcriptional expression has been recently reported in nucleated rainbow trout [31,32] and chicken [33] RBCs. However, the role of RBCs in viral antigen presentation is unknown. APCs are classified as professional or atypical [34]. Professional APCs constitutively express MHC molecules, possess machinery to process antigens, and can localize to tissues and T cell zones, whereas atypical APCs up-regulate MHC expression under certain conditions. Little evidence exists regarding atypical APCs priming T cells in an antigen-specific manner [34].

The aim of this study was to elucidate whether APCs cell markers regulation occurred in nucleated teleost RBCs after VHSV exposure, while also analyzing potential autophagy and UPS implications. These processes have been reported to generate peptides used by MHC molecules for antigen presentation [35]. Recently, we found that RBCs are nonpermissive to VHSV infection [7], but the cause of this abortive infection is being studied [9]. Our results show an increase in ubiquitination and autophagy activation in ex vivo VHSV-exposed RBCs. Inhibition of autophagy degradation led to increased levels of VHSV in RBCs. We also detected p62 degradation at early stages post infection. We found up-regulation of MHCI, MHCII, CD83, and CD86 molecules at the protein level on rainbow trout RBCs after VHSV exposure. Therefore, we show for the first time to our knowledge that nucleated RBCs can display and up-regulate APCs cell markers and process viral antigens through autophagy.

## 2. Materials and Methods

### 2.1. Animals

Rainbow trout (*Oncorhynchus mykiss*) individuals of approximately 5 to 20 gr. were obtained from a commercial fish farm (Piszolla S.L., Cimballa Fish Farm, Zaragoza, Spain). Fish were maintained at the University Miguel Hernandez (UMH) facilities in a recirculating dechlorinated water system at a stocking density of 1 fish/3L and fed daily with a commercial diet (Skretting, Burgos, Spain). Water temperature was constantly monitored to maintain fish at 14 °C. Fish were acclimatized to laboratory conditions for 2 weeks before experimentation. Experimental protocols and methods of the experimental animals were reviewed and approved by the Animal Welfare Body and the Research Ethics Committee at the UMH (approval number 2014.205.E.OEP; 2016.221.E.OEP) and by the competent authority of the Regional Ministry of Presidency and Agriculture, Fisheries, Food and Water supply (approval number 2014/VSC/PEA/00205). All methods were carried out in accordance with the Spanish Royal Decree RD 53/2013 and EU Directive 2010/63/EU for the protection of animals used for research experimentation and other scientific purposes.

### 2.2. Cell Cultures and Virus

Rainbow trout were sacrificed by overexposure to tricaine methanesulfonate (Sigma-Aldrich, Madrid, Spain) at 0.3 g/L. Peripheral blood was sampled from the caudal vein using insulin syringes (NIPRO, Bridgewater, NJ, USA). Approximately 100 µL of blood was diluted in RPMI-1640 medium (Dutch modification) (Gibco, Thermo Fischer Scientific Inc., Carlsbad, CA, USA) supplemented with 10% fetal bovine serum (FBS, Cultek, Madrid, Spain), 1 mM pyruvate (Gibco), 2 mM l-glutamine (Gibco), 50 µg/mL gentamicin (Gibco), 2 µg/mL fungizone (Gibco), and 100 U/mL penicillin/streptomycin (Sigma-Aldrich). Then, RBCs were purified by two consecutive density gradient centrifugations with Histopaque 1077 (7206g, Ficoll 1.007; Sigma-Aldrich). Finally, RBCs were washed twice with RPMI 2% FBS. An RBC purity of 99.99% was estimated by optical microscopy evaluation. Then, purified RBCs were cultured in the above indicated medium at a density of 10^7^ cells/mL in cell culture flasks at 14 °C overnight.

For autophagy assays, RBCs were treated with niclosamide (Sigma-Aldrich) after three hours post-exposure (hpe) to VHSV and then incubated for the time and at the concentration indicated for each assay. Similarly, the proteasome inhibitor MG132 (Sigma-Aldrich) was added after three hpe to VHSV and then incubated for the time and at the concentration indicated for each assay.

Viral hemorrhagic septicemia virus (VHSV-07.71) [36] was purchased from the American Type Culture Collection (ATCC, VR-1388) and propagated in fathead minnow epithelioma papulosum cyprini EPC cells (ATCC, CRL-2872) at 14 °C, as previously reported [37].

### 2.3. Antibodies

To label VHSV, we used the mouse monoclonal 2C9 antibody against the N protein of VHSV (NVHSV) [38] produced at Dr Coll’s laboratory. To label MHCI, mouse anti-MHCI against zebrafish MHCI (Ab-Mart, Shangai, China; Ref nº #X1-K4HVT2) was used (Supplementary Figure S1). Sequence alignment between zebrafish (UniprotKB Entry K4HVT2) and rainbow trout (NCBI Entry AAG53681.1) MHCI protein sequences, using NCBI BLAST tool (https://blast.ncbi.nlm.nih.gov), resulted in 48% identity and 68% positives. To label LC3, rabbit anti-LC3A/B antibody (Cell Signaling Technology, Danvers, MA; Ref nº #4108) was used. To label p62, we used rabbit anti-p62/SQSTM1 antibody (www.antibodiesonline.com; Ref nº #ABIN2854836) (Supplementary Figure S2). This antibody shows reactivity with zebrafish. Sequence alignment between zebrafish (UniprotKB Entry F1Q5Z8) and rainbow trout (XP_021439759.1) p62/sequestosome 1 protein sequences, resulted in 61% identity and 70% positives. To label ubiquitin, rabbit anti-ubiquitin antibody (StressMarq, Victoria, Canada; Ref nº #SPC-119) was used. This antibody shows reactivity with rainbow trout. Mouse anti-MHCII, mouse anti-CD86, and rabbit anti-CD83 antibodies against respective rainbow trout molecules were produced at the laboratory of Dr Luis Mercado using synthetic epitopes from the indicated molecules [39]. Western blots of anti-MHCII, anti-CD86, and anti-CD83 antibodies in RBCs can be found in Supplementary Figure S3. A polyclonal antibody against VHSV G glycoprotein (GVHSV) produced in rabbit [40], kindly donated by Dr Niels Lorenzen to Dr Julio Coll, was used in the DuoLink proximity assay. Rabbit polyclonal antibody against human α-actin (Sigma-Aldrich, Nº #2066) was used for western blotting as a loading control. Secondary antibodies used are indicated in each assay.

### 2.4. Viral Exposure Assays

Ex vivo rainbow trout RBCs were exposed to VHSV at different multiplicities of infection (MOI), as indicated in each figure. After three hours of incubation at 14 °C, cells were washed with cold RPMI, then RPMI 2% FBS was added and the culture was incubated at 14 °C for the different times indicated in each assay. Virus was not removed in the time-course assays. MOI was calculated using the following formula:

$$\mathrm{MOI}\;=\;\frac{\mathrm{Viral}\;\mathrm{titer}\;\left(\frac{{\mathrm{TCID}}_{50}}{\mathrm{mL}}\right)\cdot \mathrm{Volume}\;\mathrm{of}\;\mathrm{infection}\;(\mathrm{mL})\cdot \mathrm{Dilution}}{N{}^{\circ}\;\mathrm{of}\;\mathrm{RBCs}}$$

### 2.5. Rainbow Trout Challenge with VHSV

Young rainbow trout individuals were infected by intramuscular injection of 50 µL RPMI 2% FBS medium with VHSV (10^8^ TCID_50_/mL). As a negative control, individuals were injected with 50 µL of sterile RPMI 2% FBS. Over the course of the challenge, individuals were maintained at 14 °C for the number of days indicated. 

### 2.6. Proteasome Activity Assay

RBC proteasome activity was measured using Proteasome 20S Activity Assay Kit (Sigma-Aldrich). RBCs were exposed to VHSV for 24 h at the indicated MOI. After, approximately 2 × 10^5^ cells in 90 µL RPMI were adhered to a transparent 96-well plate previously treated with poly-D lysine (Sigma-Aldrich) by centrifugation at 800 rpm for two minutes. Then, 100 µL of Proteasome Assay Loading Solution (prepared following manufacturer instructions) were added to each well. After five hours of incubation at room temperature with protection from light, fluorescence was measured using POLARstar Omega Microplate Reader (BMG Labtech, Ortenberg, Germany) with an excitation wavelength of 490 nm and an emission wavelength of 525 nm.

### 2.7. RNA Isolation and cDNA Synthesis

The E.Z.N.A. Total RNA Kit (Omega Bio-Tek Inc., Norcross, GA, USA) was used for total RNA extraction in accordance with the manufacturer’s instructions. To eliminate possible residual genomic DNA, the sample was treated using TURBO™ DNase (Ambion, Thermo Fischer Scientific Inc.) following the manufacturer’s instructions. RNA was quantified with a NanoDrop Spectrophotometer (Nanodrop Technologies, Wilmington, DE, USA).

cDNA was synthesized from RNA using M-MLV reverse transcriptase (Invitrogen, Thermo Fischer Scientific Inc.) as previously described [41]. cDNA was stored at −20 °C.

### 2.8. Transcriptome Analysis

Ficoll-purified rainbow trout RBCs were exposed to VHSV as described above. After 4 and 72 hpe, VHSV-exposed (n = 16) and unexposed (n = 16) RBCs (10^6^ cells per fish) were resuspended in a 1/10 dilution of 9.5 μL of 10× lysis buffer (Clontech, Takara Bio, Mountain View, CA, USA) and 0.5 µL of RNase Inhibitor (Invitrogen, ThermoFisher Scientific, Waltham, MA, USA). Fish samples were grouped into 2 pools of 8 individuals for each condition (control and VHSV-exposed) and preserved at −80 °C until cDNA library construction. cDNA was directly produced from pooled lysed cells using SMART-Seq v4 Ultra Low Input RNA Kit (Clontech, Takara Bio) [31]. Sequence reads are available at SRA-NCBI accession SRP133501. RNA-Seq library preparation, sequencing, and mapping were carried out by STABVida Lda (Caparica, Portugal) as previously described [31]. 

### 2.9. Proteome Analysis

Ficoll-purified rainbow trout RBCs were exposed to VHSV as described above. At 72 hpe, VHSV-exposed (n = 16) and unexposed (n = 16) RBCs (8 × 10^6^ cells per fish) were pelletized by centrifugation (1600 rpm), the supernatant was removed, and the cell pellet was washed three times with phosphate- buffered saline (PBS), digested, cleaned-up/desalted and grouped into 2 pools of 8 individuals for each condition (control and VHSV-exposed). Then, samples were subjected to liquid chromatography and mass spectrometry analysis (LC-MS) as previously described [31]. Log_2_ peptide ratios followed a normal distribution that was fitted using least squares regression. Mean and standard deviation values derived from the Gaussian fit and were used to estimate *P* values and false discovery rates (FDR) at quantitation level. The confidence interval for protein identification was set to <95% (*P* < 0.05), and only peptides with an individual ion score above the 1% FDR threshold were considered correctly identified. Only proteins with at least two peptide spectrum matches (PSMs) were considered in the quantitation.

### 2.10. Pathway Enrichment Analysis 

Using the transcriptomic and proteomic results, differentially expressed genes (DEGs) and proteins (DEPs) pathway enrichment analyses were performed using ClueGO [42], CluePedia [43], and Cytoscape [44]. The Gene Ontology (GO) Immune System Process, GO Biological Process, Reactome pathways, KEGG pathways, and Wikipathways databases were used. A *P* value ≤ 0.05 and Kappa score of 0.4 were used as threshold values. Genes and proteins were identified by sequence homology with *Homo sapiens* using Blast2GO version 4.1.9 (BioBam, Valencia, Spain) [45].

### 2.11. Semi-quantitative PCR

Semi-quantitative PCR was performed using the commercial kit GoTaq G2 DNA polymerase (Promega, Madison, WI, USA) and synthesized cDNA. PCR reactions were performed in a total volume of 12.5 µL using 10 µM for dNTPs (Invitrogen), 0.75 mM MgCl_2_ (Promega), 1X GoTaq Green Buffer (Promega) and 1.25 U of GoTaq G2 DNA polymerase (Promega). Primer concentration was 50 nM for *cd83*, *mhcI*, and *mhcII* and 25 nM for *cd86*. A total of 12 ng of cDNA was used for each sample. Cycling conditions were 95 °C for 5 min; 35 cycles at 95 °C for 30 s, 60 °C or 62 °C (depending on the T_m_ of primers) for 30 s, and 72 °C for 20 s; and 72 °C for 5 min. An Aeris (ESCO, Singapore, Singapore) thermal cycler was used for PCR. Primers sequences used are listed in Table 1. Samples were stored at −20 °C until analysis in agarose gel electrophoresis. 

### 2.12. Agarose Gel Electrophoresis

Each amplified DNA fragment generated by semi-quantitative PCR was separated via agarose gel (2%) (Cleaver Scientific, Warwickshire, UK) electrophoresis. Gel was prepared by diluting agarose in tris-borate-EDTA buffer (TBE) (45 mM TrisHCl, 0.45 M boric acid, 10 mM EDTA) (Merck, Ñuñoa, Chile) buffer with the pH adjusted to 8. To visualize DNA bands, 0.5 µL of GelRed (Biotium, Fremont, CA, USA) were added to 25 mL of TBE buffer/agarose, and 3 µL of each sample were loaded to the gel. Electrophoresis was done at 90V for 40 min using a PowerPac 300 power supply (Biorad, CA, USA). DNA bands were visualized using UV light in an Infinity 115 (Vilber Lourmart, Marné La Vallée, France) gel documentation system with the BioCapt software (Vilber Lourmart, Marné La Vallée, France). To determine the molecular weight, we used AccRuler 100 Bp Plus DNA RTU ladder (Maestrogen, Hsinchu City, Taiwan) which includes band sizes from 3000 bp to 100 bp.

### 2.13. Gene Expression by RT-qPCR

cDNA was synthetized as previously described. RT-qPCR was performed in 20 μL reactions using 12 ng of cDNA, 10 μL of TaqMan universal PCR master mix (Thermo Fischer Scientific), 900 nM final concentration of each primer (300 nM for NVHSV gene) and 300 nM of probe (150 nM for NVHSV gene) using the ABI PRISM 7300 System (Thermo Fischer Scientific). Cycling conditions were 50 °C for 2 min; 95 °C for 10 min; and 40 cycles of 95 °C for 15 s and 60 °C for 1 min. Gene expression was analyzed by the 2^−ΔCt^ or 2^−ΔΔCt^ method [47]. The eukaryotic 18S rRNA gene (Cat#4310893E, Thermo Fischer Scientific) was used as an endogenous control. Primer and probe sequences are listed in Table 2.

### 2.14. Extracellular Immunofluorescence Staining

To stain the cell surface markers MHCI, MHCII, CD83, and CD86, RBCs were fixed in 4% paraformaldehyde (PFA; Sigma-Aldrich) and 0.008% glutaraldehyde (Sigma-Aldrich) diluted in RPMI medium for 20 min. Primary antibodies were diluted in PBS at 1/200 dilution for anti-MHCI, 1/200 for anti-MHCII, 1/100 for anti-CD83, and for 1/200 anti-CD86. Samples were incubated for 60 min. For flow cytometry, goat anti-rabbit IgG (H+L) CF™ 488 antibody (Sigma-Aldrich) was used for the secondary antibody for anti-CD83, and goat anti-mouse IgG (H+L) CF™ 488 antibody (Sigma-Aldrich) was used for anti-MHCI, anti-MHCII, and anti-CD86. Secondary antibodies were incubated for 30 min at 1/200 dilution. RBCs were washed with PBS after each antibody incubation. Flow cytometry analysis was done in a BD FACSCanto™ II (BD Biosciences) flow cytometer. Immunofluorescence (IF) images were taken with the INCell Analyzer 6000 cell imaging system (GE Healthcare, Little Chalfont, UK).

### 2.15. Intracellular Immunofluorescence Staining

RBCs were fixed with 4% PFA and 0.008% glutaraldehyde diluted in RPMI medium. RBCs were incubated with permeabilization buffer containing 0.05% saponin (Sigma-Aldrich) in PBS, for 15 min. Primary antibodies were used at 1/1000 dilution for 2C9 anti-NVHSV, 1/200 for anti-p62, and 1/100 for anti-ubiquitin in permeabilization buffer. Samples were incubated for 60 min at room temperature. Secondary antibodies were incubated for 30 min at 1/200 dilution in permeabilization buffer. RBCs were washed with permeabilization buffer after antibody incubations. Goat anti-rabbit IgG (H+L) CF™ 647 antibody and goat anti-mouse IgG (H+L) CF™ 488 antibody was used as secondary antibodies (Sigma-Aldrich). For anti-ubiquitin and anti-NVHSV double staining, goat anti-rabbit IgG (H+L) CF™ 488 antibody and goat anti-mouse IgG (H+L) CF™ 647 antibody was used as secondary antibodies. RBCs were maintained in 1% PFA in PBS. Nuclear staining was performed by staining RBCs with 1 μg/mL of 4′-6-408 Diamidino-2-phenylindole (DAPI; Sigma-Aldrich) for five minutes. 

For LC3 staining, RBCs were fixed using 4% PFA and 0.008% glutaraldehyde (Sigma-Aldrich) in PBS for 20 min and permeabilized with cold methanol (Panreac) for 15 min. LC3 antibody was diluted 1/100 in 0.3% Triton X-100 in PBS and incubated for two hours at room temperature for flow cytometry and overnight at 4 °C for immunofluorescence. Secondary antibody goat anti-rabbit IgG (H+L) CF™ 488 (Sigma-Aldrich) was diluted 1/200 in 0.3% Triton X-100 (Sigma-Aldrich) in PBS and incubated for 30 min for flow cytometry and 90 min for immunofluorescence, both at room temperature. RBCs were kept in 1% PFA in PBS before the analysis. Immunofluorescence images were taken in the INCell Analyzer 6000 cell imaging system (GE Healthcare).

### 2.16. Transmission Electron Microscopy (TEM)

Control and VHSV-exposed RBCs were fixed with glutaraldehyde at 2% in 0.1 M cacodylate buffer for three to four hours at room temperature. Post-fixation was performed with osmium tetroxide at 1% in 0.1 M cacodylate buffer for one hour at 4 °C. RBCs were centrifuged at 1600 rpm and washed with 0.1 M cacodylate buffer over 10 min three times after both steps. For the last wash, RBCs were kept at 4 °C overnight. The sample was applied to 3% agar and dehydrated using an increasing gradient of alcohol (30%, 50%, 70%, 96% and 100% during 10 min), acetone (two 10-min rounds), acetone/epon resin 1:1 (1 h), and epon resin (overnight with the Eppendorf tape open and then closed for four hours). Finally, a block with the sample was polymerized at 58 °C to 60 °C for 24 h. Images were taken using the electronic transmission microscope Jeol 1011 (JEOL, Inc. Peabody, MA, USA) from the UMH Institute of Bioengineering.

### 2.17. In situ Proximity Ligation Assay (PLA)

Superfrost microscope slides were cleaned using ethanol. Two areas of 1 cm^2^ were delimited using a Dako pen (Agilent, Santa Clara, CA, USA) on each microscope slide, and Dako pen stain dried overnight. Ficoll-purified RBCs were washed three times, and approximately 2.5 × 10^5^ RBCs were used from unexposed or VHSV-exposed (MOI 10) RBCs, at 14 °C for 24 h. RBCs were added to each area in a volume of 125 µL of RPMI. RBCs were left to sediment for 15 min. Then, RPMI was carefully removed, and 100 µL fixation buffer consisting of RPMI with 4% PFA was added for 1 h at room temperature. RBCs were washed three times with PBS after removing the fixation buffer. Then, 70% ethanol was applied to the slides for 30 s. Slides dried on ice for one hour and then were stored at −20 °C. 

Duolink In Situ–Fluorescence kit (Sigma-Aldrich) was used following the manufacturer’s instructions to perform the PLA. Once slides dried, blocking solution was added to each area, and slides were incubated for one hour at 37ºC in a wet chamber. Blocking solution was removed, and a mixture containing the primary antibodies mouse anti-MHCI (1/200) or anti-MHCII (1/200) and polyclonal rabbit anti- GVHSV antibody [40] (kindly provided by Dr Neils Lorenzen to Dr Julio Coll) (1/300) were incubated overnight in a wet chamber at 4 °C. Alternatively, anti-MHCI or anti-MHCII were incubated together with rabbit serum (1/300) to detect nonspecific background signals. After incubation with the primary antibodies, RBCs were washed twice with wash buffer A for 5 min with slow agitation. Excess wash buffer A was removed, and the PLA Probes MINUS reagent was incubated at a 1/5 dilution for 1 h at 37 °C in the wet chamber. Then, ligation shock reagent and ligase were added to the RBCs after washing. Amplification reagents were added to the RBCs and then removed after 100 min of incubation. Slides were mounted with a cover slip using DuoLink In Situ Mounting Medium with DAPI and stored at −20 °C until analysis.

To quantify positive colocalization between MHCI or MHCII and GVHSV peptides in RBCs, we used a counting algorithm in the IN Cell Developer software (GE Healthcare). Briefly, RBC cytoplasm was delimited using a collar around the nucleus (labeled by DAPI) of a ~5 µm radius. Positive colocalization was noted by detection of granules inside the RBCs cytoplasm (settings were adjusted for a minimum brightness and granular size to be considered for colocalization between the two molecules). 

### 2.18. Western Blot

RBCs pellets (10^7^ cells) and head kidney tissue samples were resuspended in 100 µL PBS buffer with a protease inhibitor cocktail (Sigma-Aldrich). Cells were lysed by freezing and thawing samples three times. Tissues were disrupted using micropestles (Invitrogen). Cell debris was eliminated by centrifugation at 12,000 rpm for 10 min. Samples were loaded in a 12% polyacrylamide gel (Invitrogen), except for anti-ubiquitin which was at 16%, under reducing conditions. Electrophoresis was performed at 150 V for 100 min. Proteins in the gel were transferred to 0.4 µm pore size nitrocellulose membranes (BioRad, Madrid, Spain) for 120 min at 100 V in transfer buffer (2.5 mM Tris, 9 mM glycine, 20% methanol). Membranes were then blocked with 5% dry milk and 0.2% Tween-20 in PBS and incubated with rabbit polyclonal anti-ubiquitin, rabbit polyclonal anti-αactin (42 kDa), mouse monoclonal anti-MHCI (45 kDa), mouse polyclonal anti-MHCII (~34 kDa), mouse polyclonal anti-CD86 (~31 kDa), or rabbit polyclonal anti-CD83 (~24 kDa) in PBS containing 5% dry milk and 0.2% Tween-20 (blocking buffer) overnight at 4 °C. Membranes were washed three times for 10 min each with PBS Tween-20 0.2% buffer before incubation with GAR-Po (Sigma-Aldrich) or GAM-Po (Sigma-Aldrich) in blocking buffer for 60 min. Membranes were then washed three times with PBS Tween-20 0.2%. Peroxidase activity was detected using enhanced chemiluminescence (ECL) reagents (Amersham Biosciences, Buckinghamshire, UK) and exposure to X-ray. Protein lanes and bands were analyzed by densitometry using ImageJ software (version 1.51, National Institutes of Health, Bethesda, MD, USA). Lanes were selected using the rectangle tool of ImageJ software and the integrated density of the lane was measured. α-actin band densitometry was calculated by plotting the band density after selecting the bands with the rectangle tool.

### 2.19. Software and Statistics

All graphs show the mean and standard deviation of the data. *P* values associated with each graphic are represented by: *, *P* value < 0.05; **, *P* value < 0.01; ***, *P* value < 0.001; ****, *P* value < 0.0001. Graphpad Prism 6 (www.graphpad.com) (Graphpad Software Inc., San Diego, CA, USA) was used to prepare graphs and perform statistical calculations. Flow cytometry data were analyzed using Flowing Software v2.5.1 (http://flowingsoftware.btk.fi/) to obtain mean fluorescence intensity (MFI) values and Weasel v3.0.1 (https://frankbattye.com.au/Weasel/) to obtain graphical representation of histograms and dot plots. 

## 3. Results

### 3.1. Transcriptomic Analysis Indicated Up-Regulation of Antigen-Processing-Related Molecules in Ex Vivo VHSV-Exposed Rainbow Trout RBCs

**Figure 1:**
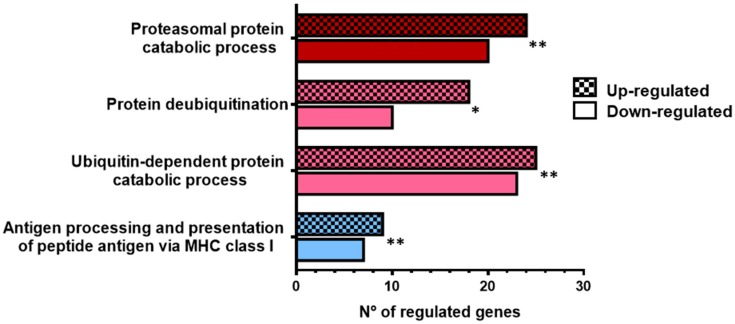
Transcriptomic analysis indicated up-regulation of antigen-processing-related molecules in ex vivo VHSV-exposed rainbow trout RBCs. Number of up-regulated and down-regulated genes related to proteasomal protein and catabolic process (GO:0010498), protein deubiquitination (GO:0016579), ubiquitin-dependent protein catabolic process (GO:0006511), antigen processing and presentation of peptide antigen via MHC class I (GO:0002474) (Supplementary Table S1), by RNA-Seq from ex vivo unexposed and VHSV-exposed RBCs at 4 hpe. Asterisks denote GO-term significance.

To identify major processes activated when rainbow trout RBCs are exposed to VHSV, a transcriptomic analysis using RNA-Seq and pathway enrichment evaluation were performed on VHSV-exposed RBCs at 4 and 72 hpe. Several up-regulated genes were classified into GO categories of ubiquitination and proteasome degradation and MHC class I antigen processing and presentation (Figure 1, Supplementary Table S1) at 4 hpe. Selected genes belonging to the ubiquitination and proteasome degradation category are listed in Table 3 (Supplementary Tables S1 and S2). Among these up-regulated genes are cullin 3 (*cul3*) and proteasome subunits α6 (*psma6*) and β5 (*psmb5*). Also related to the MHCI presentation pathway, our analysis identified calnexin (*canx*), GTPase activating protein SEC13 (*sec13*), and inhibitor of nuclear factor kappa B kinase (*ikbkb*). Ras-related rab 7 (*rab7*) and tumor necrosis factor (TNF) receptor-associated factor 6 (*traf6*) were analyzed by RT-qPCR as genes related to the MHCI presentation pathway. RT-qPCR validation of the genes identified in the transcriptomic analysis is shown in Supplementary Figure S4, where a tendency to up-regulation is observed at 4 hpe, although the RT-qPCR data do not strongly support the fold changes found by RNA-Seq. Moreover, we also identified up-regulation of some genes involved in autophagy, such as unc-51–like autophagy activating kinase 1 (*ulk1*), beclin 1 (*becn1*), and autophagy-related 9A (*atg9a*) (Table 4). In contrast, at 72 hpe, RBCs showed a global down-regulation (Supplementary Tables S1 and S2).

### 3.2. Proteomic Analysis of VHSV-Exposed RBCs Showed Proteasome Down-Regulation, Increased Ubiquitination, and Regulation of Antigen Presentation-Related Molecules at 72 hpe

**Figure 2:**
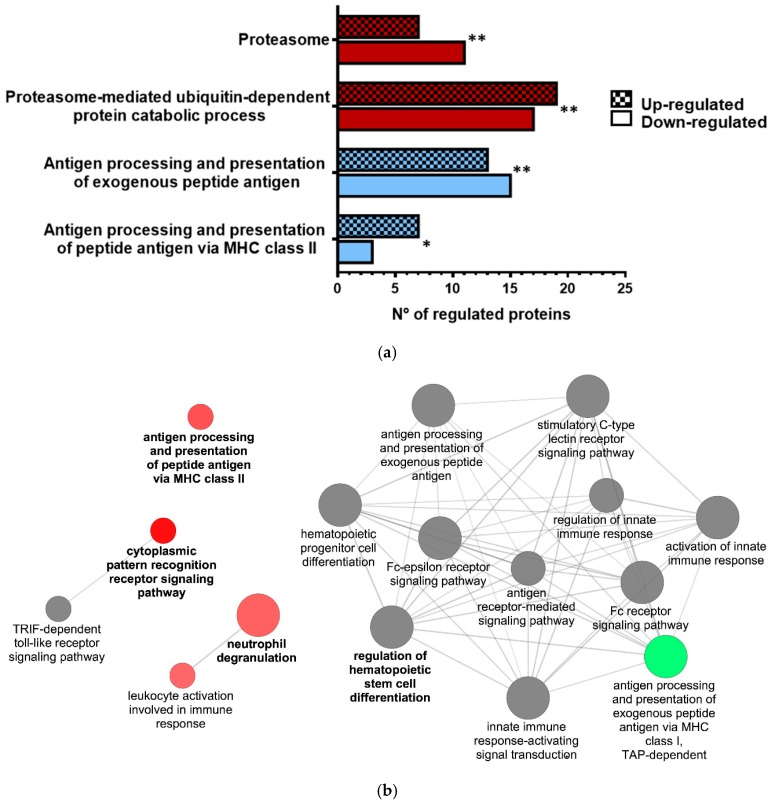
Proteomic analysis of VHSV-exposed RBCs showed proteasome down-regulation, increased ubiquitination, and regulation of molecules from antigen presentation pathways at 72 hpe. (**a**) Number of up-regulated and down-regulated proteins related to proteasome (KEGG:03050), proteasome-mediated ubiquitin-dependent protein catabolic process (GO:0043161), antigen-processing and presentation of exogenous peptide antigen (GO:0002478), and antigen processing and presentation of peptide antigen via MHC class II (GO:0002495), as identified by proteomic analysis from ex vivo unexposed and VHSV-exposed rainbow trout RBCs at 72 hpe (Supplementary Table S3). Asterisks denote GO-term significance. (**b**) Cytoscape pathway network of significantly overrepresented Immune System Process GO terms in VHSV-exposed RBCs at 72 hpe (Supplementary Table S3). Each node represents a GO-term from GO Immune System Process. Node size shows GO-term significance (*P* value): a smaller *P* value indicates larger node size. Edge (line) between nodes indicates the presence of common genes: a thicker line implies a larger overlap. The label of the most significant GO-term for each group is highlighted. Up-regulated pathways are coded as red, while down-regulated pathways are coded as green. Pathways with a similar number of up-regulated or down-regulated proteins are coded as gray. Asterisks denote statistical significance.

We analyzed the response of ex vivo RBCs to VHSV at 72 hpe using a proteomic analysis and pathway enrichment evaluation. Up-regulated proteins were overrepresented in antigen processing and presentation of peptide antigen via MHC class II (GO:0002495), and proteasome-mediated ubiquitin-dependent protein catabolic process (GO:0043161), while proteasome (KEGG:03050) and antigen-processing and presentation of exogenous peptide antigen (GO:0002478) were mostly down-regulated (Figure 2a). A list of all overrepresented terms and statistics is provided in Supplementary Table S3. Table 5 displays the fold change of proteins from these categories (Supplementary Table S4).

A Cytoscape pathway network of significantly overrepresented Immune System Process GO terms showed up-regulation in antigen processing and presentation of peptide antigen via MHC class II, cytoplasmic pattern recognition receptor signaling pathway, neutrophil degranulation, and leukocyte activation; however, it showed down-regulation of antigen presentation via MHC class I. (Figure 2b). In Supplementary Figure S5, we show a Venn diagram to compare the common products found in our omics studies.

### 3.3. VHSV Induced Ubiquitination But Impaired Proteasome Degradation in Ex Vivo VHSV-exposed Rainbow Trout RBCs

**Figure 3:**
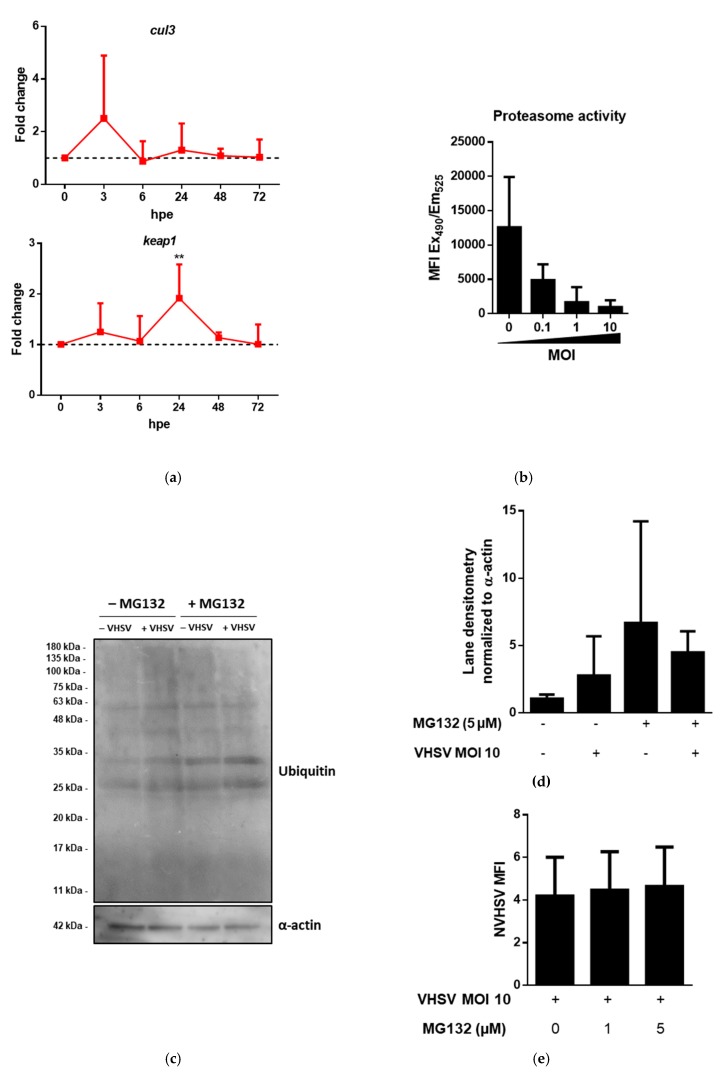

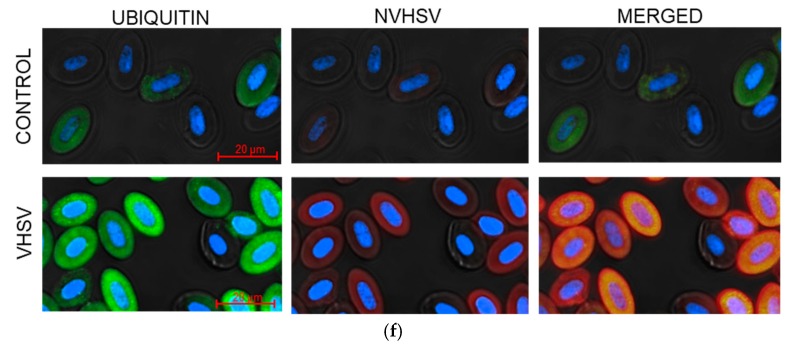
VHSV induced protein ubiquitination but impaired proteasome degradation in ex vivo VHSV-exposed rainbow trout RBCs. (**a**) Time-course expression of *cul3* and *keap1* at 0, 3, 6, 24, 48, and 72 hpe from VHSV-exposed (MOI 1) RBCs. Data represent mean ± SD (n = 5), relative to control cells (black dotted line). A two-way analysis of variance (ANOVA) with Sidak´s multiple comparisons test was performed to test statistical significance between unexposed and VHSV-exposed RBCs at each time point. (**b**) 20S proteasome activity measured by fluorogenic substrates in RBCs unexposed or exposed to VHSV at the indicated MOI at 24 hpe. Data represent mean ± SD (n = 3). Kruskal-Wallis with Dunn’s multiple comparisons test was performed to test statistical significance between each condition and unexposed RBCs. (**c**) Western blot of ubiquitin of lysates from unexposed and VHSV-exposed (MOI 10) RBCs at 24 hpe, treated with or without MG132 (5 µM). α-actin was used as endogenous control. Results are representative of 2 independent experiments. (**d**) Integrated densitometry of ubiquitin lane content from unexposed and VHSV-exposed (MOI 10) RBCs at 24 hpe, treated with or without MG132 (5 µM). Values were normalized to α-actin. Data represent mean ± SD (n = 2). (**e**) Intracellular quantification by flow cytometry of NVHSV in VHSV-exposed (MOI 10) RBCs at 72 hpe, treated with or without MG132 (1 or 5 µM). Data represent mean ± SD (n = 4). Kruskal-Wallis with Dunn’s multiple comparisons test was performed to test statistical significance between MG132 treated and non-treated RBCs. (**f**) Representative immunofluorescence of unexposed (control) and VHSV-exposed RBCs at MOI 100 and at 9 hpe stained with anti-ubiquitin (488 stain), 2C9 anti-NVHSV (647 stain), and DAPI for nuclei staining. Asterisks denote statistical significance.

To validate the role of the UPS in the nonpermissive infection of rainbow trout RBCs by VHSV, we performed a time-course experiment analyzing the expression of two genes belonging to the ubiquitin E3 ligase complex: *cul3* and kelch-like ECH-associated protein 1 (*keap1*). The results showed increased expression of *cul3* at 3 hpe while *keap1* expression increased at 12 hpe (Figure 3a). We measured the activity of the 20S proteasomes using a commercial kit and observed a MOI-dependent decrease in 20S proteasome activity (Figure 3b). Then, we performed a western blot using an anti-ubiquitin antibody for unexposed and VHSV-exposed RBCs with or without the proteasome inhibitor MG132. Ubiquitination of proteins on VHSV-exposed RBCs increased in comparison with unexposed RBCs. A higher amount of ubiquitinated proteins was also found in RBCs treated with MG132 (Figure 3c,d). To test whether the proteasome is involved in the degradation of VHSV, we assessed the presence of NVHSV using 2C9 monoclonal antibody in VHSV-exposed RBCs treated with MG132. Flow cytometry results did not show an increase in intracellular NVHSV in VHSV-exposed RBCs treated with MG132 (Figure 3e). The population used for the flow cytometry analysis is depicted in Supplementary Figure S6. Double staining using 2C9 and anti-ubiquitin antibodies showed higher ubiquitin labeling in RBCs with VHSV (Figure 3f).

### 3.4. VHSV Induced Autophagy in Ex Vivo VHSV-exposed Rainbow Trout RBCs

**Figure 4:**
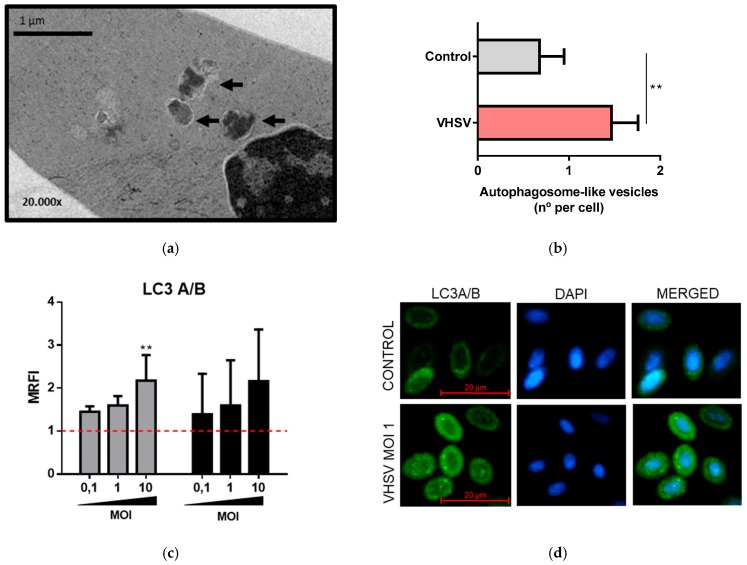

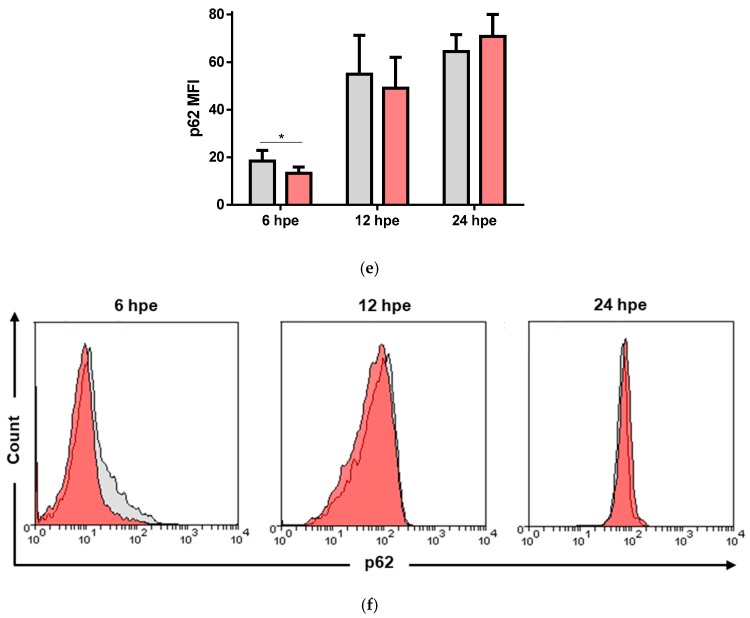
VHSV induced autophagy in ex vivo VHSV-exposed rainbow trout RBCs. (**a**) Representative transmission electron micrographs of VHSV-exposed RBCs, pointing out autophagosome-like vesicles (black arrows). (**b**) Count of autophagosome-like vesicles from transmission electron micrographs of unexposed and VHSV-exposed rainbow trout. Data represent the mean ± SD (n = 30). A Mann-Whitney test was performed to test statistical significance. (**c**) Autophagosome membrane protein LC3 expression levels in VHSV-exposed RBCs at 24 (gray bars) and 72 hpe (black bars) relative to unexposed RBCs (red line) evaluated by flow cytometry (n = 5). Data is represented as MRFI (Mean Relative Fluorescence Intensity) = fluorescence in VHSV-exposed RBCs/fluorescence in non-exposed RBCs. A Kruskal-Wallis with Dunn´s multiple comparisons test was performed to test statistical significance between each condition and unexposed RBCs. (**d**) Representative immunofluorescence of unexposed (control) and VHSV-exposed RBCs at MOI 1 and at 72 hpe stained with anti-LC3 (488) and DAPI for nuclei staining. (**e**) Mean fluorescence intensity of p62 protein expression in RBCs unexposed (gray bars) and exposed to VHSV at MOI 10 (red bars) after 6, 12, and 24 hpe. Data represent the mean ± SD (n = 5). A Mann-Whitney test was performed to test statistical significance between unexposed and VHSV-exposed RBCs at each time point. (**f**) Representative histograms of p62 in RBCs exposed to VHSV (MOI 10) at 6, 12, and 24 hpe: unexposed (gray histogram), VHSV-exposed (red histogram). Asterisks denote statistical significance.

To determine whether VHSV induced autophagy in ex vivo rainbow trout RBCs, we exposed RBCs to VHSV at MOI 1 for 24 h. We identified the presence of autophagosome-like vesicles inside VHSV-exposed RBCs (Figure 4a) via TEM. We visually counted the number of autophagosome-like vesicles in unexposed RBCs and VHSV-exposed RBCs and noted a significant increase in VHSV-exposed RBCs (Figure 4b). The turnover of the autophagy protein LC3A/B was monitored by means of LC3A/B immunostaining, as previously described for rainbow trout cells [14,51]. LC3 immunostaining increased at higher MOIs in a dose-dependent manner up to 2-fold in comparison with unexposed RBCs at 24 and 72 hpe (Figure 4c). By immunofluorescence microscopy, we identified an increased number of LC3 dots in VHSV-exposed RBCs (Figure 4d). Moreover, we analyzed the ubiquitin-binding protein p62, which undergoes degradation during activation of autophagy [52], as it is an autophagosome cargo protein that targets other proteins for selective autophagy. To evaluate whether p62 undergoes degradation in the RBC response to VHSV, an intracellular staining using anti-p62 antibody was performed on unexposed and VHSV-exposed (MOI 10) RBCs at 6, 12, and 24 hpe. Decreased intracellular p62 levels were detected in VHSV-exposed RBCs at 6 hpe compared to control RBCs (Figure 4e,f). By 24 hpe, p62 levels recovered from the degradation observed at 6 hpe. Kinetics of expression of the autophagy-related genes *ulk1*, *becn1* and *gabarap* showed statistically significant up-regulation at 3 hpe (Supplementary Figure S7).

### 3.5. Niclosamide Increased p62 and Intracellular VHSV Levels in Ex Vivo VHSV-exposed RBCs

**Figure 5:**
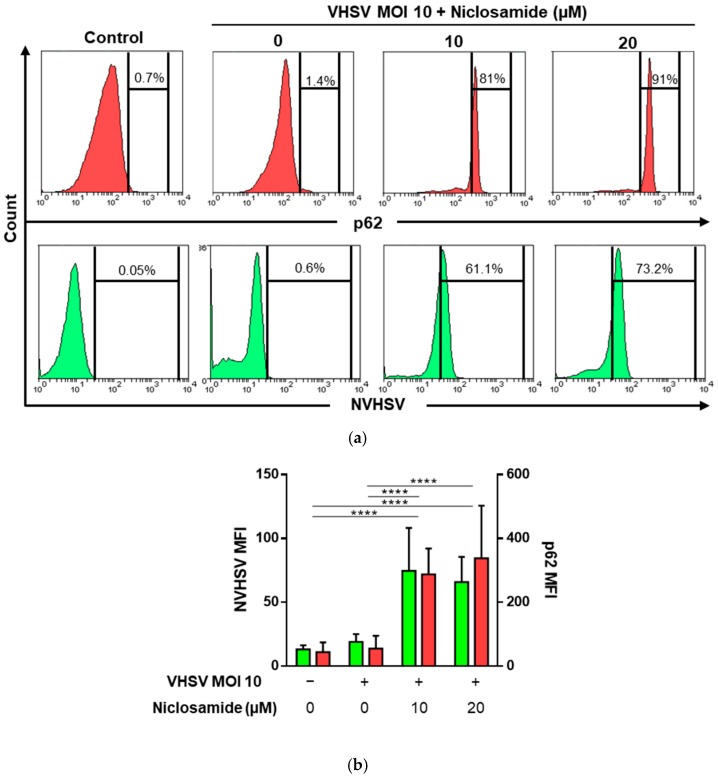
Niclosamide increased p62 and intracellular VHSV levels in ex vivo VHSV-exposed RBCs. (**a**) Representative histograms of NVHSV (green) and p62 (red) intracellular expression in RBCs unexposed (control) and VHSV-exposed (MOI 10) RBCs treated or not with niclosamide 10 or 20 µM and evaluated by flow cytometry at 72 hpe. Percentages represent the number of positive cells. Dimethyl sulfoxide (DMSO) was added to untreated RBCs to match culture conditions of treated cells (DMSO 0.04%). (**b**) MFI of intracellular NVHSV (green) and p62 (red) in unexposed (control) and VHSV-exposed (MOI 10) RBCs treated or not with niclosamide 10 or 20 µM and evaluated by flow cytometry at 72 hpe. Data represent mean ± SD (n = 6). A two-way ANOVA with Tukey´s multiple comparisons test was performed to test statistical significance. Asterisks denote statistical significance.

The drug niclosamide blocks autophagy degradation via lysosomal dysfunction [53,54]. Moreover, niclosamide has been previously used in the context of viral infections [55]. After exposing RBCs to VHSV MOI 10, RBCs were treated with niclosamide at 10 and 20 µM. Then, an intracellular stain using 2C9 and anti-p62 antibodies was done at 72 hpe. Flow cytometry results showed that VHSV-exposed RBCs treated with niclosamide at both tested concentrations had a higher percentage of NVHSV- and p62-positive cells compared to RBCs exposed to VHSV but not treated with niclosamide (Figure 5a). MFI of unexposed RBCs and VHSV-exposed (MOI 10) RBCs were similar, but both NVHSV and p62 MFI increased up to three-fold in the presence of niclosamide (Figure 5b). 

### 3.6. Rainbow Trout RBCs Up-Regulated MHCI, MHCII, CD86, and CD83 after VHSV Exposure

**Figure 6:**
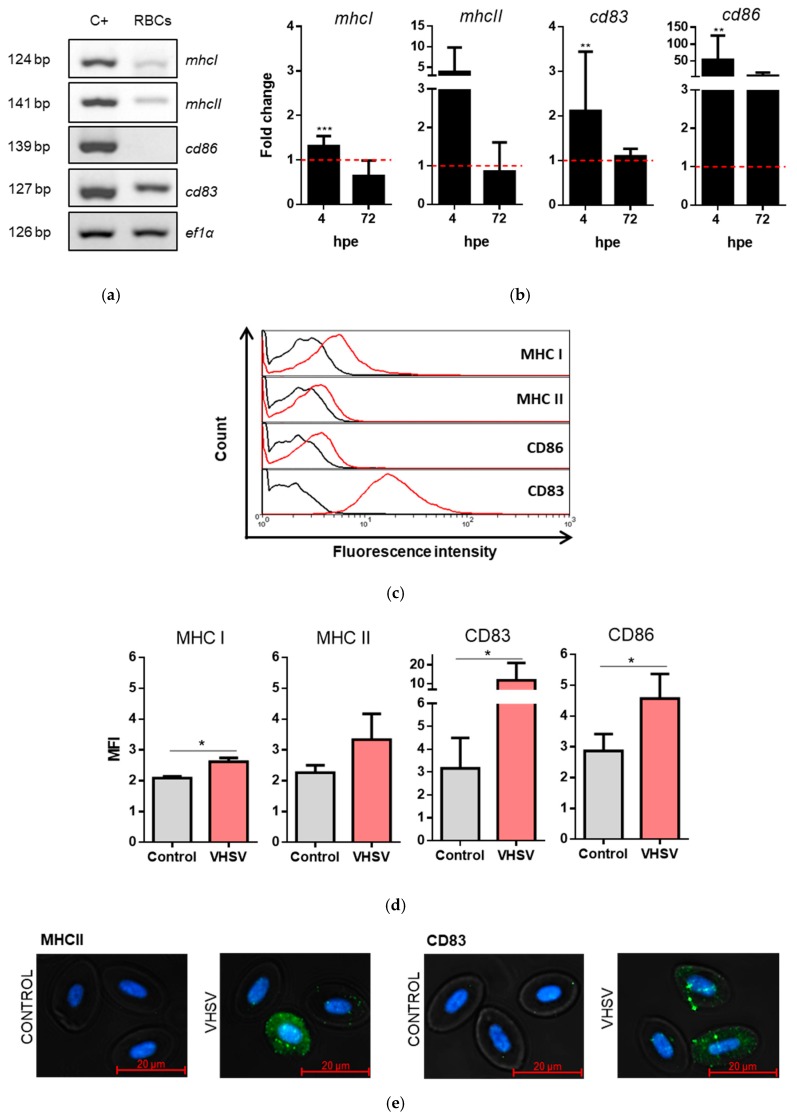
Rainbow trout RBCs up-regulated MHCI, MHCII, CD83, and CD86 molecules after exposure to VHSV. (**a**) Specific transcript mRNA expression of *mhcI*, *mhcII*, *cd86*, and *cd83* genes from rainbow trout RBCs. A mix of gill, spleen, and head kidney tissues was used as a positive control of expression from the assayed cell markers (C+). The *ef1α* gene was used as an endogenous control. (**b**) Fold change in the expression of *mhcI*, *mhcII*, *cd86*, and *cd83* in rainbow trout RBCs at 4 and 72 hpe with VHSV MOI 1 in comparison to unexposed RBCs, by RT-qPCR. Data represent mean ± SD (n = 4). Dotted red line represents basal gene expression from unexposed RBCs. A Mann-Whitney test was performed to test statistical significance between VHSV-exposed and unexposed RBCs. (**c**) Representative histograms of MHCI, MHCII, CD86, and CD83 extracellular stain in unexposed RBCs (black) and VHSV-exposed RBCs (red) (MOI 10) at 24 hpe. (**d**) MFI of MHCI, MHCII, CD86, and CD83 extracellular stain in unexposed RBCs (gray bars) and VHSV-exposed RBCs (red bars) (MOI 10) at 24 hpe. Data represent mean ± SD (n = 4). A Mann-Whitney test was performed to test statistical significances between VHSV-exposed and unexposed RBCs. (**e**) Representative immunofluorescence images of MHCII and CD83 expression in control and VHSV-exposed RBCs at 24 hpe. Asterisks denote statistical significance.

Because antigen presentation pathways were overrepresented in transcriptomic and proteomic analyses, we investigated whether RBCs expressed characteristic cell markers molecules of APCs. RNA was extracted from RBCs and then we performed RT-PCR. Semi-quantitative PCR was performed, and a mix of tissue samples from the head kidney, spleen, and gill was used as a positive control for APCs genes expression. Final products from semi-quantitative PCR were analyzed in agarose gel electrophoresis. mRNA transcripts from *mhcI*, *mhcII*, and *cd83* were detected in rainbow trout RBCs, whereas there was no *cd86* expression (Figure 6a). We then examined how VHSV modified the expression of these transcripts using quantitative RT-qPCR. We observed a slight increase in *mhcI* expression and a pronounced increase in *mhcII*, *cd83*, and *cd86* expression in VHSV-exposed RBCs at 4 hpe. Whereas, we only observed up-regulation of *cd86* at 72 hpe (although lower than levels at 4 hpe), while no up-regulation was observed at 72 hpe for the *mhcI*, *mhcII,* and *cd83* genes (Figure 6b). We confirmed the up-regulation of MHCI, MHCII, CD86, and CD83 at the protein level in VHSV-exposed RBCs 24 hpe (Figure 6c–e). Also, *cd83* gene expression was found to be up-regulated by transcriptomic analysis at 4 hpe (Log_2_ fold: 4.87; Supplementary Table S2). In contrast, MHCI protein expression was found down-regulated by proteomic analysis at 72 hpe (Log_2_ fold: −11.38; Supplementary Table S4). 

### 3.7. VHSV Induced Autophagy and Antigen Presentation Genes Expression in RBCs from VHSV-challenged Rainbow Trout

**Figure 7:**
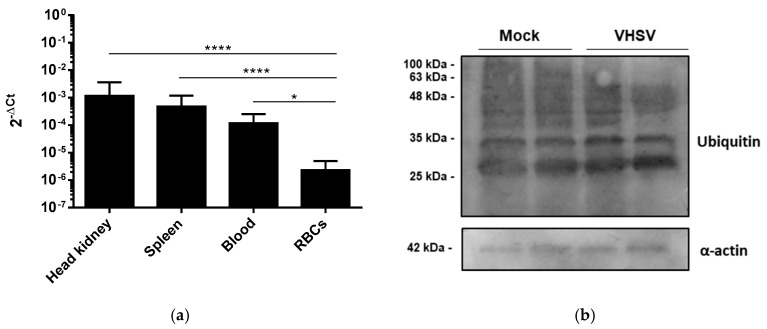

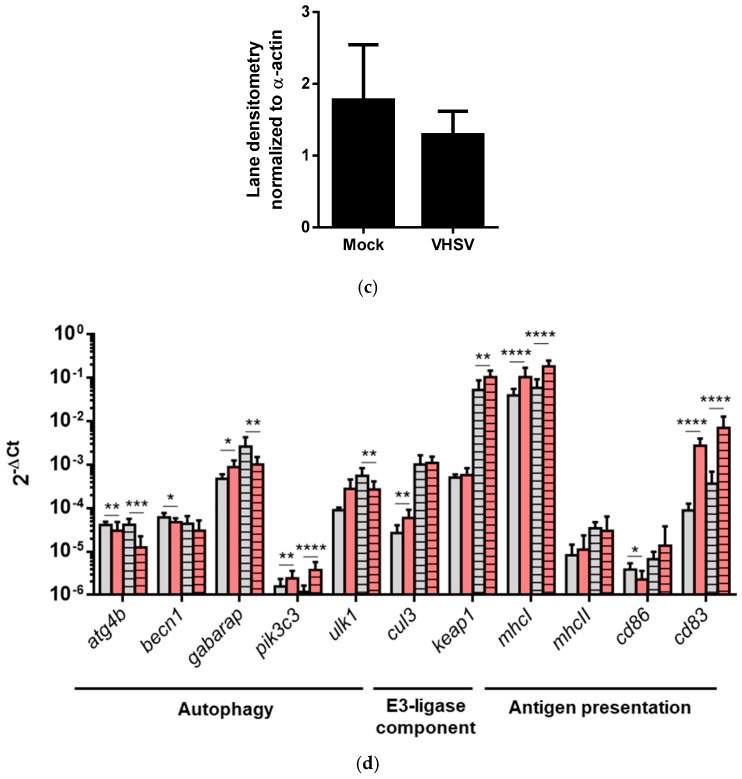
VHSV induced autophagy, E3 ubiquitin ligase components, and antigen presentation genes expression in RBCs from VHSV-challenged rainbow trout. (**a**) Quantification of *NVHSV* in head kidney, spleen, blood, and purified RBC samples from challenged rainbow trout 2 dpc. Data represent mean ± SD (n = 7). A Kruskal-Wallis with Dunn´s multiple comparisons test was performed to test statistical significance. (**b**) Western blot of ubiquitin in RBCs from mock and VHSV-challenged rainbow trout at 2 dpc. α-actin was used as a loading control. Samples from 2 individuals were loaded for each condition. (**c**) Densitometry bar plot of ubiquitin lane protein content of RBCs from mock and VHSV-challenged rainbow trout after 2 dpc. Values were normalized to α-actin. Data represent mean ± SD (n = 2). Mann-Whitney test was used to test statistical differences. (**d**) Gene expression values of the autophagy-related genes *atg4b*, *ulk1*, *becn1*, *gabarap*, and *pik3c3*; E3 ligase component genes *cul3* and *keap1;* and antigen presentation genes *mhcI*, *mhcII*, *cd83*, and *cd86* measured by RT-qPCR in RBCs from mock (gray) and VHSV-challenged (red) rainbow trout at 1 dpc (no pattern) and 2 dpc (striped pattern). Data represent mean ± SD (n = 6). A Mann-Whitney test was performed to test statistical significances between RBCs from mock and VHSV-challenged rainbow trout. Asterisks denote statistical significance.

We evaluated whether VHSV could induce both autophagy- and antigen-presentation-related genes in vivo by using RBCs from VHSV-challenged and mock-infected rainbow trout. We used tissue samples from the spleen and head kidney, as well as total blood and Ficoll-purified RBC samples, from VHSV-challenged and mock-infected rainbow trout to quantify NVHSV gene transcripts by RT-qPCR. RBCs from challenged rainbow trout showed lower levels of NVHSV in comparison with total blood, spleen, and head kidney samples (Figure 7a). We also analyzed ubiquitination of proteins in RBCs from VHSV-challenged and mock-infected rainbow trout and we did not observe an increase in ubiquitinated proteins at 2 days post challenge (dpc) (Figure 7b,c), in contrast to ex vivo experiments. We analyzed the expression of a set of genes related to autophagy, E3 ubiquitin ligase component, and antigen presentation in RBCs from VHSV-challenged rainbow trout after 1 and 2 dpc by RT-qPCR. The expression of autophagy-related genes *gabarap* and *pik3c3* was significantly up-regulated at 1 dpc. However, only *pik3c3* gene expression was observed up-regulated at 2 dpc. On the other hand, *atg4b* and *becn1* genes were down-regulated at 1 and 2 dpc. *ulk1* gene expression was also down-regulated at 2 dpc (Figure 7d). For E3 ubiquitin ligase components, *cul3* and *keap1* expression was significantly increased at 1 and 2 dpc, respectively (Figure 7d). For antigen presentation-related genes, *mhcI* and *cd83* were highly up-regulated in RBCs from VHSV-challenged rainbow trout at 1 and 2 dpc, while *cd86* was significantly down-regulated at 1 dpc. *mhcII* gene expression showed a tendency to increase at 1 dpc, but not significantly (Figure 7d).

### 3.8. GVHSV Protein Peptides Colocalize with MHCI and MHCII in VHSV-Exposed Rainbow Trout RBCs

**Figure 8:**
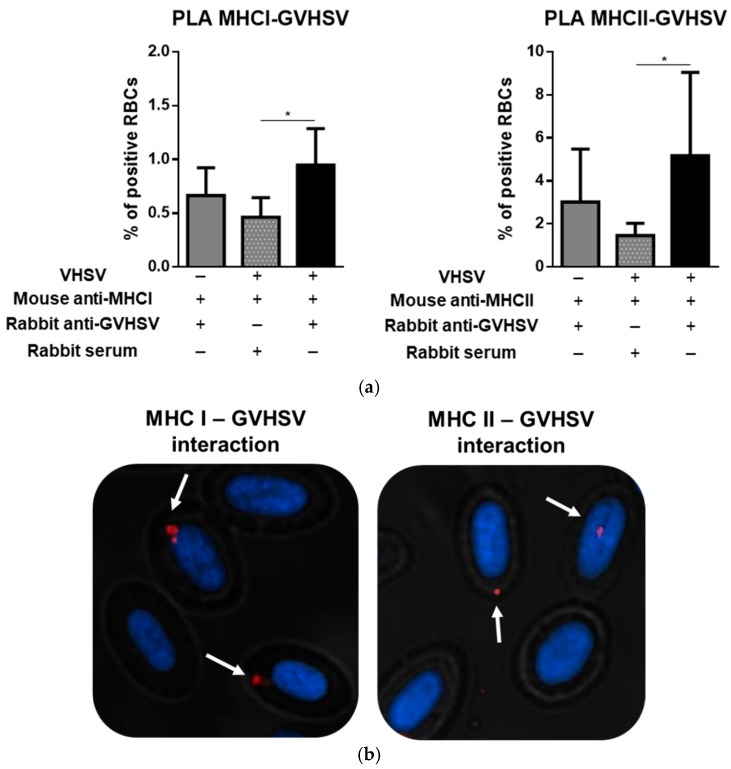
GVHSV protein peptides colocalize with MHCI and MHCII in VHSV-exposed rainbow trout RBCs. (**a**) Percentages of positive RBCs in MHCI – GVHSV and MHCII – GVHSV interaction under unexposed and VHSV-exposed conditions. Rabbit serum was used to test unspecific interaction with mouse anti-MHCI and anti-MHCII antibodies in VHSV-exposed RBCs. Data represent percentage of positives RBCs counted by IN Cell Developer software using an algorithm to detect fluorescent events in RBCs (n = 2 individuals, 8 fields were analyzed in each slide). A Kruskal-Wallis with Dunn´s multiple comparisons test was performed to test statistical significances between all the conditions. (**b**) Representative microscopy images of Duolink PLA for MHCI or MHCII and GVHSV in VHSV-exposed RBCs. Positive RBCs for the PLA are indicated with white arrows. Asterisks denote statistical significance.

To establish a correlation between the presence of VHSV peptides from autophagy and MHCI and MHCII molecules (the expression of which was up-regulated after VHSV exposure), we performed a PLA between MHCI or MHCII and VHSV using the DuoLink kit. At 24 hpe, RBCs were stained using a rabbit polyclonal antibody against GVHSV and a mouse monoclonal antibody against MHCI or MHCII. We observed an increase in the percentage of positive cells in VHSV-exposed RBCs in contrast to unexposed RBCs (Figure 8a). A representative positive colocalization is shown in Figure 8b.

## 4. Discussion

**Figure 9:**
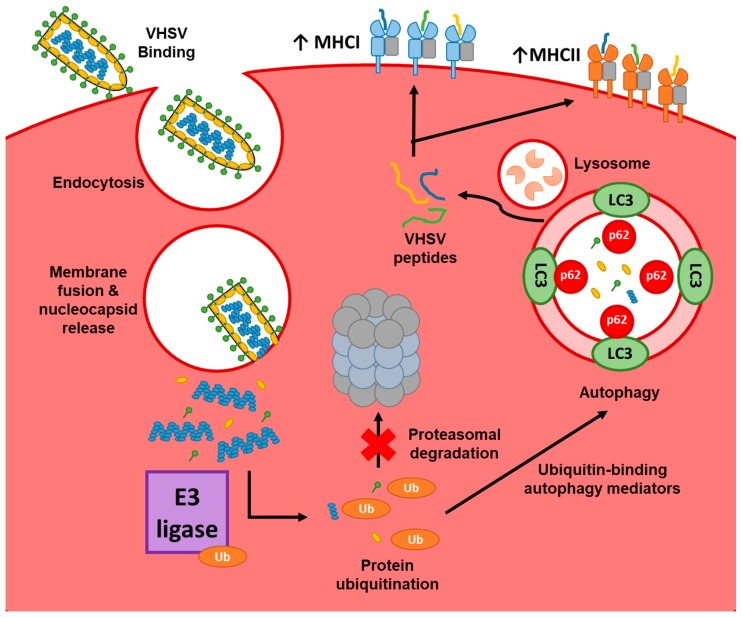
Proposed schematic representation of processes involved in VHSV degradation and antigen processing in rainbow trout nucleated RBCs. VHSV cell entry is mediated by endosome acidification, which leads to membrane fusion and thus release of the capsid. RBC transcription of autophagy genes and components of the E3 ubiquitin ligase then starts and intracellular proteins are ubiquitinated to be marked for degradation. The low proteasome activity induced as a consequence of the VHSV proteins presence leads to the accumulation of ubiquitinated proteins that are suggested to be degraded in the autophagosome. Finally, peptides from this process can be coupled into MHC molecules that are later transported to the membrane to potentially participate in the antigen presentation process.

In this study, we have demonstrated that autophagy is implicated in the clearance of VHSV virions in nucleated rainbow trout RBCs, a cell whose main known function has been oxygen transportation. While previous studies have identified virus-related autophagy in teleost RBCs [16] and have localized the expression of MHCI molecules to the surface of nucleated RBCs [30], even in different vertebrate species [56], our results provide the first evidence of nucleated RBCs up-regulating APC markers in the context of a viral infection. Our findings suggest that RBCs could potentially play a new role in which autophagy is involved in viral protein degradation and the generated peptides are coupled to MHC molecules. A graphical summary of this process is shown in Figure 9.

Transcriptomic analysis of RBCs at four hours after VHSV exposure showed up-regulation of *cul3*, *keap1*, *psma6*, and *psmb5* genes from the antigen-processing category. *cul3* and *keap1* are components of the E3 ubiquitin ligase complex involved in the ubiquitination of proteins targeted for proteasome degradation [57], and *psma6* and *psmb5* are part of proteasome complexes. In the MHCI presentation pathway, our analysis identified *canx*, which is involved in the assembly of MHCI [58]; *sec13*, whose expression correlates with the expression of MHCI [58]; and *ikbkb*. These results correlated with the increase in ubiquitinated proteins induced by VHSV as detected by western blot. Different viruses have been reported to induce ubiquitination. This effect was observed with West Nile Virus; its capsid protein was ubiquitinated by Makorin ring finger 1 protein and later sent for proteasome degradation [59]. Ubiquitination was also reported for the core protein of human hepatitis C virus [60] and turnip yellow mosaic virus [61]. However, our results also showed lower proteasome activity, which could be due in part to the accumulation of ubiquitinated proteins in VHSV-exposed RBCs. Proteasome activity has been reported to favor the replication of different viruses [62,63], and it has been found to prevent viral replication [64]. In contrast, proteasome activity did not seem to play a role in VHSV degradation in our study. 

Increased autophagy activity was demonstrated both at the transcriptional and translational levels in VHSV-exposed RBCs. Several studies have shown a protective role of autophagy against different viruses, including dengue [65], sindbis [66], vesicular stomatitis virus (VSV) [67], and VHSV [14]. Our results demonstrated that VHSV exposure induced autophagy in rainbow trout RBCs; this prevented VHSV infection, as shown by p62 degradation and the results observed when VHSV-exposed RBCs were treated with niclosamide, which led to the accumulation of both p62 and VHSV. p62 accumulation suggested that autophagosome degradation was blocked in RBCs. We also observed an increase in intracellular VHSV. Therefore, autophagy may be a mechanism involved in VHSV degradation. We previously reported that the N:G gene expression ratio in RBCs exposed to VHSV was lower than commonly reported ratio levels [7], indicating that VHSV replication in RBCs was inhibited early after VHSV exposure. VHSV starts replication of the N gene within the first 6 hpe in RBCs in the permissive cell line RTG2 [7], so all processes aiming to inhibit VHSV infection in RBCs should occur during this time. Our results correlated with this report, since there was an early transcriptional response of autophagy-related genes together with p62 degradation at 6 hpe. p62 has been described to be the link between autophagy and the UPS [68,69], since autophagosome degradation of ubiquitinated proteins has been already reported [70], and p62 itself undergoes degradation upon autophagy activation. Moreover, some studies have reported a direct interaction between p62 and different viruses [66] or bacteria [71]. Our results showed a decrease in p62 levels that later recovered [72], suggesting that p62 may act as an adaptor protein that targeted VHSV for autophagic degradation, although this cannot be confirmed because we have not observed interaction between p62 and VHSV proteins. In this sense, other ubiquitin-binding autophagy mediators such as NRB1, NDP52 and optineurin [73] could be evaluated in the future.

RBCs exhibited an APC-like profile with MHCII, CD86, and CD83 endogenous expression. CD86 and CD83 are known costimulatory cell surface markers of APC maturation [74] and are involved in the regulation of different immune processes, such as lymphocytes proliferation and activation [75]. The presence of MHCII with the costimulatory molecules CD83 and CD86 suggests a more professionalized APC profile for RBCs, since MHCI has been reported to be expressed in almost all nucleated cells [76]. Our results showed modulation in the expression of MHCI, MHCII, CD83, and CD86 proteins when RBCs were exposed to VHSV. Antigen presentation via MHCI is normally associated with peptides derived from UPS, but recent reports have shown a contribution of autophagy to antigen presentation via MHCI molecules [77]. On the other hand, autophagy is the main source of peptides for MHCII molecules [78]. Moreover, we showed that antigen presentation via MHCI and MHCII potentially could be functional, because peptides from GVHSV colocalized with MHC molecules. Recently, it has been reported that different cell types called atypical APCs, such as neutrophils [79] or lymph node stromal cells [80] could be involved in antigen presentation, supporting the hypothesis that these atypical APCs could play an important role in various immune processes apart from antigen presentation [34]. However, to properly classify teleost RBCs as a typical APC, studies are needed to test their ability to activate naïve T cells, as this is main difference between atypical and typical APCs [34].

The results obtained from ex vivo RBCs culture experiments were partially corroborated under an in vivo scenario. RBCs from VHSV-challenged rainbow trout showed lower *NVHSV* transcript load compared to other tissues, similar to VHSV halted infection in ex vivo RBCs cultures [7]. We observed the expression of autophagy genes early after VHSV challenge, similar to the kinetics observed in ex vivo RBCs exposed to VHSV. In addition, we observed up-regulation of *cul3* at 1 dpc followed by *keap1* up-regulation at 2 dpc, just as we observed in ex vivo time-course experiments. In vivo results showed up-regulation of *mhcI*, *mhcII*, *cd83*, and *cd86* in RBCs from challenged rainbow trout, which correlated with their increased expression observed in ex vivo RBCs exposed to VHSV. In contrast, lack of ubiquitination was observed in RBCs from VHSV-challenged rainbow trout. 

In summary, after VHSV cell entry into RBCs, the transcription of autophagy genes and components of the E3 ubiquitin ligase started. The low proteasome activity that was induced as a consequence of the presence of VHSV led to the accumulation of ubiquitinated proteins. Finally, peptides from this process could be coupled into intracellular MHC molecules that would be later transported to the membrane to potentially participate in the antigen presentation process. Further studies are being performed to fully describe the potential functional APC role in nucleated teleost RBCs to ascertain how MHC molecules participate or are implicated in the presentation of degraded viral antigens in nucleated RBCs. Given that RBCs are the most abundant cell type in the blood, this new knowledge will shed light on the design of novel vaccine targets. Potential applications of these results could imply that RBCs, which can be transfected and induce immune gene expression [32], are target of new strategies for vaccination.

## Figures and Tables

**Table 1:** List of primer sequences used for semi-quantitative PCR.

Gene	Forward Primer (5′–3′)	Reverse Primer (5′–3′)	Reference or Accession Number
*mhcI*	CCAGAGGATGTATGGTTGTGAG	TGGAGCGATCCATGTCTTTGTC	AF287490.1
*mhcII*	GTACTCCAGGTGGGAGTGGA	TGCAGCGCCTATGACTTCTA	AY273808.1
*cd86*	ATGTAACAGTGGCCTGTGA	CCACCCACTGCTGTTCACTA	FJ607781.1
*cd83*	GGAGCGTGAAGTGAACTTT	TCCTGGTTCTGCTCTCCTACA	AY263797.1
*ef1α*	TGGAGACTGGCACCCTGAAG	CCAACATTGTCACCAGGCATGG	[46]

**Table 2:** List of primers and probes sequences used in quantitative RT-qPCR.

Gene	Forward Primer (5′–3′)	Reverse Primer (5′–3′)	Probe (5′–3′)	Reference or Accession Number
*atg4b*	GATCCTGTCCCTGTGATGATGA	CCCCTATTGGCTTCCCTTCT	ACCCCCCCCGGCGATTCTTC	XR_002473879
*ulk1*	CTTCTGCTGCTGGGTCTTCTG	GGTGACGGAAGAACTCCTCAAA	CGAAACCACAAGGACCGCATGGA	XR_002473462
*becn1*	GCGTGGGTGTCGTCTCAGTT	CAGGGAAGCAAGGAGAGCAT	ACCCTGGGTGTGCCCCTTGACC	NM_001124429
*gabarap*	CCTCATCCATCCATTTTTACCTCTT	ATTCAACCGAAATCCCCATCT	TCTGAATTTTATTTGCCTCCGGGTCTCC	NM_001165091
*pik3c3*	AGGCCAGCTGTGTGTGTTTCTA	GTTGCACATAGCGTTCCTGTTTA	TTTGCCCCCCCGGATGATTGA	XM_021577851
*cul3*	GCAGCTTACGTTACAGCATCACA	TGGTGTTGGAGCCTGTTACCT	AACGCCACCTTCTACGGCCCAATC	XM_021587294.1
*ikbkb*	TGTTCCTGTTTGACCGTTCCT	CCGTCTGGACAAAGCGTATGT	CCTACGAGCCCCAGTTCACCCCC	XM_021621802.1
*keap1*	CCTCCACAAGCCCACCAA	AAGTATCCCCCTGCCGTGTA	CACGCCCAAAGTGCCCCAGC	XM_021556738.1
*rab7*	GTTGCGTGCTGGTGTTTGAC	ACTCGTCCCTCCAGCTGTCTAG	TGACCGCCCCCAACACCTTCAA	XM_021609589.1
*sec13*	GCAGTGATCCAGGCACAGAA	CTGGGACTAGGATAGATGGTAGAAGTG	ATTCCACTCCTCCTCCTACCCCCACA	XM_021610740.1
*traf6*	AGGACGCGGTGTGGAAGAG	CATGAATCTTGCTGTCCTCGTAAA	AGATGCACCAAAGCCAACACTGCCA	XM_021586866.1
*mhcI*	GACAGTCCGTCCCTCAGTGT	CTGGAAGGTTCCATCATCGT		[48]
*mhcII*	TGCCATGCTGATGTGCAG	GTCCCTCAGCCAGGTCACT	CGCCTATGACTTCTACCCCAAACAAAT	[49]
*cd86*	GGTCTGTGACCCTCCCCTGTA	CCCTCGTCTTATGGTAGCCATT		XR_002470439.1
*cd83*	TTGGCTGATGATTCTTTCGATATC	TGCTGCCAGGAGACACTTGT	TCCTGCCCAATGTAACGGCTGTTGA	[50]
*NVHSV*	GACTCAACGGGACAGGAATGA	GGGCAATGCCCAAGTTGTT	TGGGTTGTTCACCCAGGCCGC	[41]

**Table 3:** Fold change of genes from the “class I MHC-mediated antigen processing and presentation” and “antigen processing: ubiquitination and proteasome degradation” pathways in the transcriptomic analysis of VHSV-exposed rainbow trout RBCs at 4 hpe. Gene expression values were calculated by normalization against unexposed RBCs. Gene *P* values were <0.001 and FDR *P* values < 0.05. Gene symbols correspond to homologue *Homo sapiens* genes identified by sequence homology using Blast2GO.

Antigen Processing: Ubiquitination and Proteasome Degradation		Class I MHC-Mediated Antigen Processing and Presentation	
Gene Symbol	Log_2_ Fold	Gene Symbol	Log_2_ Fold
*cul3*	4.77	*canx*	4.31
*keap1*	7.56	*sec13*	5.35
*psma6*	5.02	*ikbkb*	5.69
*psmb5*	3.72	*klhl13*	5.36

**Table 4:** Fold change of the autophagy-related genes *ulk1*, *becn1*, and *atg9a* obtained in the transcriptomic analysis of VHSV-exposed rainbow trout RBCs at 4 hpe. Gene expression values were calculated by normalization against uninfected RBCs. Gene *P* values were < 0.001 and FDR *P* values < 0.05.

Autophagy-Related Genes	
Gene Symbol	Log_2_ Fold
*ulk1*	3.46
*becn1*	5.55
*atg9a*	3.69

**Table 5:** List of up-regulated (left) and down-regulated (right) identified proteins from the “antigen processing and presentation of peptide antigen via MHC class II”, “proteasome-mediated ubiquitin-dependent protein catabolic process” and “proteasome” pathways. Protein FDR *P* values were < 0.001. Protein symbols correspond to homologue *Homo sapiens* proteins identified by sequence homology using Blast2GO.

Antigen Processing and Presentation of Peptide Antigen via MHC Class II				Proteasome-mediated Ubiquitin-Dependent Protein Catabolic Process				Proteasome			
Upr. Protein	Log_2_ Fold	Downr. Protein	Log_2_ Fold	Upr. Protein	Log_2_ Fold	Downr. Protein	Log_2_ Fold	Upr. Protein	Log_2_ Fold	Downr. Protein	Log_2_ Fold
ACTR1B	3.37	CAPZB	−2.68	CD2AP	7.50	HSPA5	−6.23	PSMB3	4.44	PSMA1	−5.33
AP2S1	5.75	CLTC	−3.69	DDB1	3.32	PSMA1	−5.33	PSMB6	3.73	PSMA2	−5.43
CLTA	4.51	RAB7A	−4.69	GCLC	4.63	PSMA2	−5.43	PSMC2	2.98	PSMA3	−3.31
DNM2	5.32			HSPA1A	4.74	PSMA3	−3.31	PSMD13	2.26	PSMA4	−4.78
DYNC1H1	5.43			NPLOC4	1.68	PSMA4	−4.78	PSMD2	3.98	PSMA5	−6.15
KIF15	3.89			PLAA	5.08	PSMA5	−6.15	PSMD4	5.83	PSMA6	−6.49
PYCARD	3.28			PSMB3	4.44	PSMA6	−6.49	PSME1	5.73	PSMA8	−5.50
				PSMB6	3.73	PSMA8	−5.50			PSMB1	−4.45
				PSMC2	2.98	PSMB1	−4.45			PSMB2	−5.29
				PSMD13	2.26	PSMB2	−5.29			PSMB4	−3.69
				PSMD2	3.98	PSMB4	−3.69			PSME2	−3.44
				PSMD4	5.83	PSME2	−3.44				
				PSME1	5.73	RAD23B	−3.77				
				RACK1	3.95	UBC	−5.19				
				RAD23A	4.85	UBR2	−11.48				
				RPS27A	5.03	VCP	−2.86				
				UBB	4.39	WFS1	−10.33				
				USP19	8.29						
				YOD1	2.95						

## Supplementary Materials

The following are available online at https://www.mdpi.com/2073-4409/8/5/386/s1. Figure S1: Anti-MHCI zebrafish antibody labeling in rainbow trout. (a) Western blot of anti-MHCI in rainbow trout RTS11 monocyte/macrophage-like cell line and Ficoll-purified red blood cells (RBCs). Black arrow indicates a protein band around 45 kDa marker. (b) Immunofluorescence of anti-MHCI in rainbow trout RBCs. Figure S2: Anti-p62/SQSTM1 zebrafish antibody labeling in rainbow trout. (a) Western blot of anti-p62/SQSTM1 in rainbow trout RBCs. Black arrow indicates a protein band under 63 kDa marker. (b) Immunofluorescence of anti-p62 in VHSV-exposed rainbow trout RBCs. White arrow indicates vesicle-like dots. Figure S3: Protein expression of MHCII, CD83, and CD86 in rainbow trout head kidney (HK) and RBCs, by means of western blot. HK samples were used as positive control of expression. Figure S4. Validation of RNA-Seq study by RT-qPCR representing fold change of *cul3*, *psmb5*, *keap1*, *sec13*, *ikbkb*, *rab7*, and *traf6* genes in the transcriptomic analysis of VHSV-exposed RBCs at 4 and 72 hpe. Gene expression values were calculated with normalization to unexposed RBCs. Data represent the mean ± SD (n = 4). Mann-Whitney test was used to test statistical significances between VHSV-exposed and unexposed RBCs. Figure S5: Venn diagram of the common omics products identified by transcriptomics at 4 and 72 h post-exposure and proteomics at 72 h post-exposure. Figure S6: Gated population of RBCs used for flow cytometry analysis. Figure S7. Time-course expression of the autophagy-related genes *beclin1*, *ulk1*, and *gabarap* at 0, 3, 6, 24, 48, and 72 hpe from unexposed and VHSV-exposed (MOI 1) RBCs. Data represent the mean ± SD (n = 5) relative to control cells (black dotted line). A Two-way ANOVA with Sidak´s multiple comparisons test was performed to test statistical significance between unexposed and VHSV-exposed RBCs at each time point. Table S1: GO Terms identified in VHSV-exposed RBCs after 4 and 72 h post-exposure by RNA-Seq. Table S2: List of significantly regulated genes after 4 and 72 h post-exposure in VHSV-exposed RBCs. All the listed genes have an FDR lower than 0.05. Table S3: GO Terms identified in VHSV-exposed RBCs after 72 h post-exposure by proteomic sequencing. Table S4: List of significantly regulated proteins after 72 h post-exposure in VHSV-exposed RBCs. All the listed proteins have an FDR lower than 0.001.
