# Supplementary material for: Rainbow Trout Red Blood Cells Exposed to Viral Hemorrhagic Septicemia Virus Up-Regulate Antigen-Processing Mechanisms and MHC I&II, CD86, and CD83 Antigen-presenting Cell Markers
Source: Cells. 2019 Apr 27;8(5):386. doi: 10.3390/cells8050386 (PMC6562805; doi:10.3390/cells8050386)

Supplementary Figure S7. Time-course expression of the autophagy-related genes *beclin1*, *ulk1*, and *gabarap* at 0, 3, 6, 24, 48, and 72 hpe from unexposed and VHSV-exposed (MOI 1) RBCs. Data represent the mean ± SD (n = 5) relative to control cells (black dotted line). A Two-way ANOVA with Sidak´s multiple comparisons test was performed to test statistical significance between unexposed and VHSV-exposed RBCs at each time point.

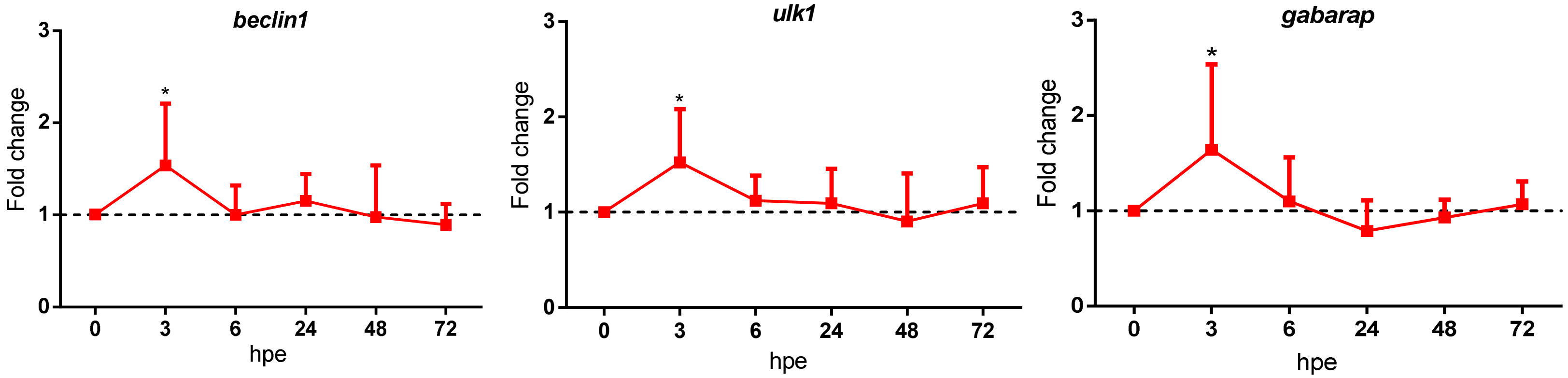

Supplement: Supplementary file 1 [file cells-08-00386-s001.zip › Supplementary Figure S7.pdf]
