# Supplementary material for: Rainbow Trout Red Blood Cells Exposed to Viral Hemorrhagic Septicemia Virus Up-Regulate Antigen-Processing Mechanisms and MHC I&II, CD86, and CD83 Antigen-presenting Cell Markers
Source: Cells. 2019 Apr 27;8(5):386. doi: 10.3390/cells8050386 (PMC6562805; doi:10.3390/cells8050386)

Supplementary Figure S1. Anti-MHCI zebrafish antibody labelling in rainbow trout. (A) Western blot of anti-MHCI in rainbow trout RTS11 monocyte/macrophage-like cell line and Ficoll-purified red blood cells (RBCs). Black arrow indicates a protein band around 45 kDa marker. (B) Immunofluorescence of anti-MHCI in rainbow trout RBCs.

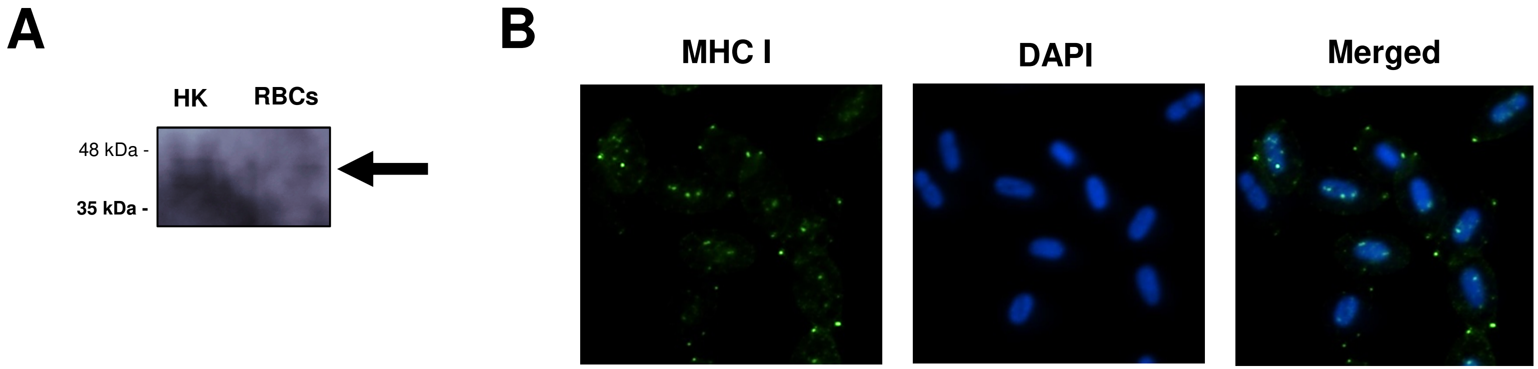

Supplement: Supplementary file 1 [file cells-08-00386-s001.zip › Supplementary Figure S1.pdf]
