# Supplementary material for: Rainbow Trout Red Blood Cells Exposed to Viral Hemorrhagic Septicemia Virus Up-Regulate Antigen-Processing Mechanisms and MHC I&II, CD86, and CD83 Antigen-presenting Cell Markers
Source: Cells. 2019 Apr 27;8(5):386. doi: 10.3390/cells8050386 (PMC6562805; doi:10.3390/cells8050386)

Supplementary Figure S3. Protein expression of MHCII, CD83, and CD86 in rainbow trout head kidney (HK) and red blood cells (RBCs), by means of western blot. HK samples were used as positive control of expression.

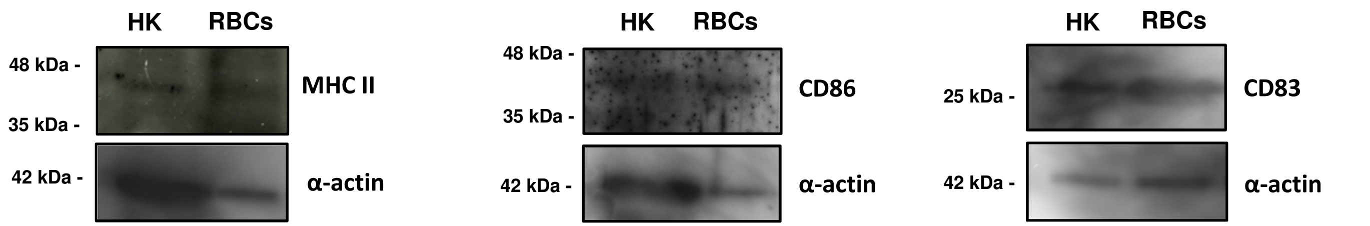

Supplement: Supplementary file 1 [file cells-08-00386-s001.zip › Supplementary Figure S3.pdf]
