# Supplementary material for: Rainbow Trout Red Blood Cells Exposed to Viral Hemorrhagic Septicemia Virus Up-Regulate Antigen-Processing Mechanisms and MHC I&II, CD86, and CD83 Antigen-presenting Cell Markers
Source: Cells. 2019 Apr 27;8(5):386. doi: 10.3390/cells8050386 (PMC6562805; doi:10.3390/cells8050386)

Supplementary Figure S4. Validation of RNASeq results by RT-qPCR representing fold change of *cul3*, *psmb5*, *keap1*, *sec13*, *ikbkb*, *rab7* and *traf6* genes in the transcriptomic analysis of VHSV-exposed RBCs at 4 and 72 hpe. Gene expression values were calculated with normalization to unexposed RBCs. Data represent the mean ± SD (n=4). A Mann-Whitney test was used to test statistical significances between VHSV-exposed and unexposed RBCs.

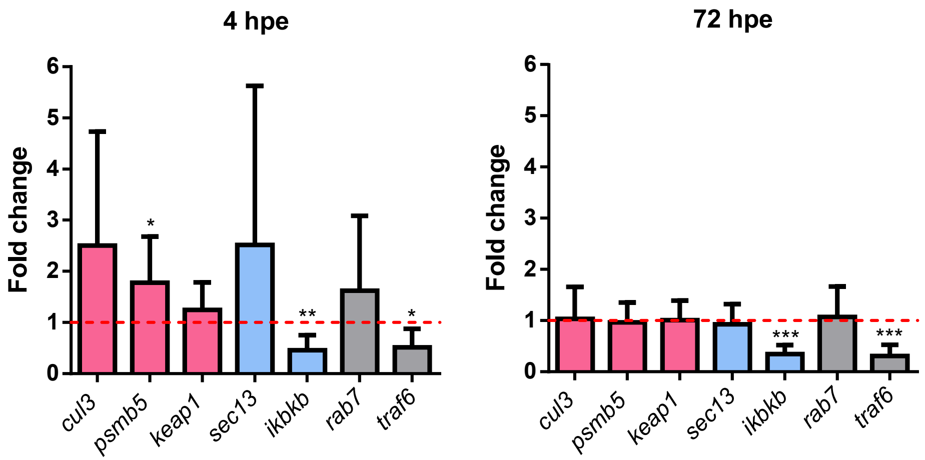

Supplement: Supplementary file 1 [file cells-08-00386-s001.zip › Supplementary Figure S4_2.pdf]
