# Supplementary material for: Rainbow Trout Red Blood Cells Exposed to Viral Hemorrhagic Septicemia Virus Up-Regulate Antigen-Processing Mechanisms and MHC I&II, CD86, and CD83 Antigen-presenting Cell Markers
Source: Cells. 2019 Apr 27;8(5):386. doi: 10.3390/cells8050386 (PMC6562805; doi:10.3390/cells8050386)

Supplementary Figure S5. Venn diagram of the common omics products identified by transcriptomics at 4 and 72 hpe and proteomics at 72 hpe.

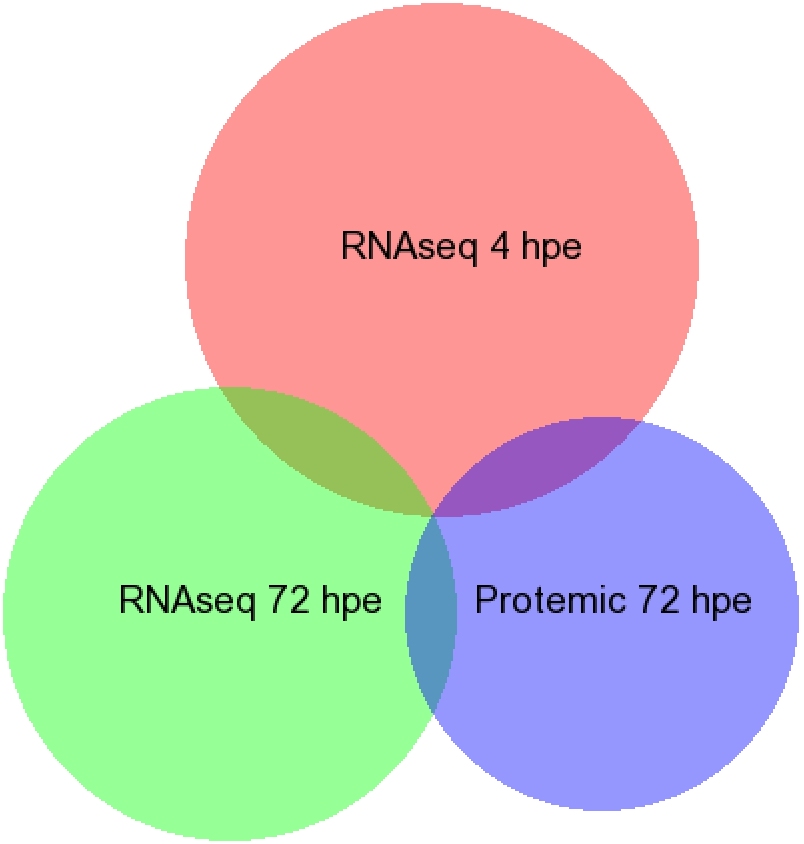

Supplement: Supplementary file 1 [file cells-08-00386-s001.zip › Supplementary Figure S5_2.pdf]
