# Supplementary figures and images for: Rainbow Trout Red Blood Cells Exposed to Viral Hemorrhagic Septicemia Virus Up-Regulate Antigen-Processing Mechanisms and MHC I&II, CD86, and CD83 Antigen-presenting Cell Markers
Source: Cells. 2019 Apr 27;8(5):386. doi: 10.3390/cells8050386 (PMC6562805; doi:10.3390/cells8050386)

**Supplementary Figure S6.** Gated population of RBCs used for flow cytometry analysis.

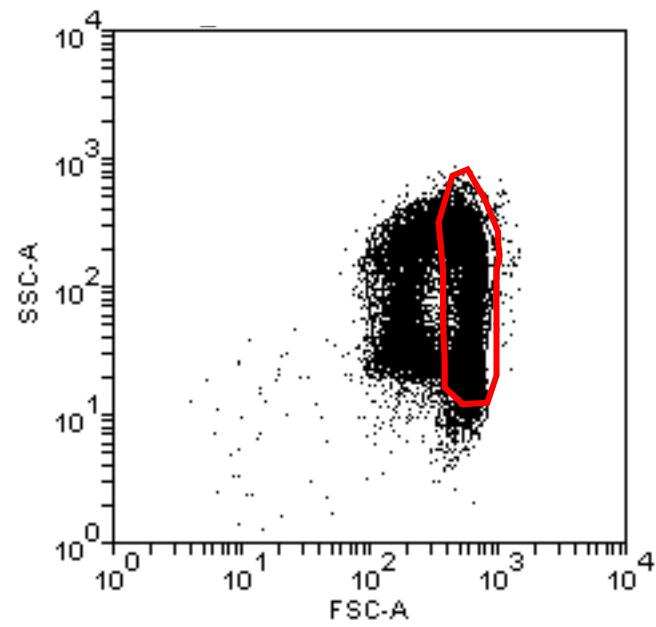

Supplement: Supplementary file 1 [file cells-08-00386-s001.zip › Supplementary Figure S6.pdf]
